# Extensive spinal epidural abscess due to *Streptococcus intermedius*: a case report treated conservatively and literature review

**DOI:** 10.3389/fneur.2023.1237007

**Published:** 2023-08-24

**Authors:** Dianqi Liu, Weijie Lu, Wenbin Huang, Wenrun Zhai, Qinjie Ling

**Affiliations:** Department of Orthopedic Surgery, The First Affiliated Hospital of Guangzhou Medical University, Guangzhou, China

**Keywords:** spinal epidural abscess, *Streptococcus intermedius*, antibiotic, decompression, magnetic resonance imaging

## Abstract

**Purpose:**

To describe the clinical significance of prompt, adequate, and targeted intravenous antibiotic (IV antibiotic) therapy in the successful management of spinal epidural abscess (SEA) associated with *Streptococcus intermedius* (*S. intermedius*) infection.

**Case description:**

SEA is a rare, but catastrophic infection that may result in a high risk of permanent neurological disability. A 52-year-old Chinese female patient was presented to the emergency department due to 2 years of low back pain and 3 days of decreased muscle strength in the extremities. The blood culture confirmed the presence of *S. intermedius* infection, and gadolinium-enhanced magnetic resonance imaging (MRI) demonstrated widespread epidural abscesses in the cervical, thoracic, and lumbar spine canal. Empirical IV antibiotic therapy with vancomycin was promptly initiated, with meropenem and moxifloxacin added subsequently based on blood culture results. After 5 days of IV antibiotic treatment, the patient’s blood culture became negative. 6 weeks later, a follow-up MRI showed a decrease in the size of the abscess. The patient’s muscle strength was mostly restored after 2 months of IV antibiotic treatment.

**Conclusion:**

Repeat examinations or gadolinium-enhanced MRI should be considered when initial MRI findings are not diagnostic of SEA. For extensive SEA caused by *Streptococcus intermedius* infection, surgery may be non-essential, and the judicious antibiotic selection and adequate treatment duration are pivotal for successful conservative management. Furthermore, for patients who are not amenable to surgery, a comprehensive evaluation of their condition and meticulous implementation of a precise pharmacological regimen holds noteworthy clinical significance.

## Introduction

Spinal epidural abscess (SEA) is a rare yet potentially devastating pyogenic injury that occurs between the spinal dura mater, posterior longitudinal ligament, and vertebral periosteum (1). Rapid abscess enlargement can lead to symptoms such as radicular pain, paralysis, and even death. Therefore, it is essential to diagnose infections early and treat them with a combination of intravenous antibiotic (IV antibiotic) and decompression surgery. Although life-threatening bacterial infections are uncommon, the clinical association of *Streptococcus intermedius* (*S. intermedius*) with abscess formation has long been recognized. *Streptococcus intermedius*, also referred to as *Streptococcus milleri* group (SMG) (2), is a member of the *Streptococcus anginosus* group (SAG). Within this group, *S. intermedius* has the capability to secrete a novel cytotoxin known as intermedilysin, which specifically targets human cells. Intermedilysin exhibits potent hemolytic activity exclusively toward human erythrocytes and no hemolysis on equine or goat blood agar, commonly employed in clinical microbiology. However, because this cytotoxin exhibits beta-hemolytic activity on human blood agar medium, *S. intermedius* is classified as a beta-hemolytic Gram-positive coccus. The first reported case of a brain abscess caused by *S. intermedius* dates back to 1975 (2). Subsequent cases have shed light on the pathogenesis of *S. intermedius*-associated abscesses in patients with congenital heart disease and sinusitis. Masalma et al. (3) conducted a study on 20 patients with brain abscesses associated with *S. intermedius* and found that this condition has several known risk factors for the development of invasive CNS disease, including endodontic infection, dental caries, and periodontitis, as confirmed by multiple 16S ribosomal DNA sequencing. To increase awareness of this rare condition, we present a case study of an extensive SEA patient with an *S. intermedius* infection, describing the clinical course, imaging characteristics, treatment, and patient prognosis.

## Case presentation

**Figure 1:**
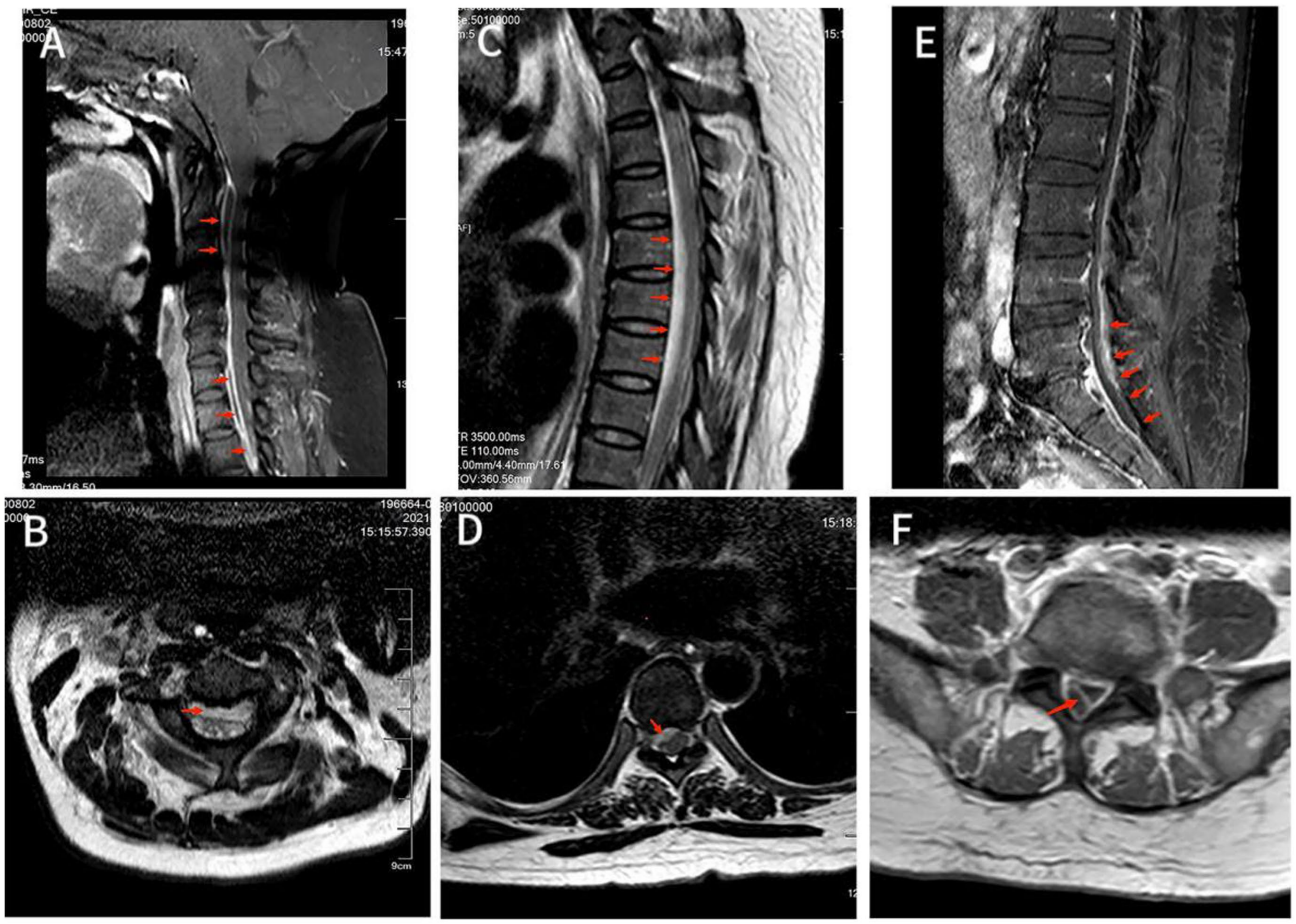
**(A)** MRI T2-weighted image showed hyperintensity of the lesion on the ventral side of the spinal cord in the C2-C6 spinal canal. Also saw C5/6 intervertebral disc herniation and the corresponding dura and spinal cord were compressed. **(B)** Axial T2-weighted image showed an annular high signal lesion in the anterior part of the spinal cord within the spinal canal of C2, and spinal cord compression at the C2. **(C)** T2-weighted image showed hyperintensity of lesions ventral to the spinal cord in the T2-T8 spinal canal, with corresponding levels of spinal cord compression degeneration. **(D)** Axial T2-weighted image showed annular high signal lesion in the right anterior part of the spinal cord within the spinal canal of T7 accompanied by nerve root and spinal cord compression. **(E)** T1-enhanced image showed disc bulging in L3/4, L4/5 and L5/S1 with annular enhancement of the lesion ventral to the spinal cord in the L4-S1 spinal canal with spinal cord compression at the corresponding level, but no significant internal enhancement. **(F)** Axial T1-enhanced image showed a large range of irregular non-enhanced areas in the soft tissue of the right posterior side of the at the L5 level, and a ring-enhancing lesion in the right posterior part of the intradural sac with no obvious internal enhancement.

**Figure 2:**
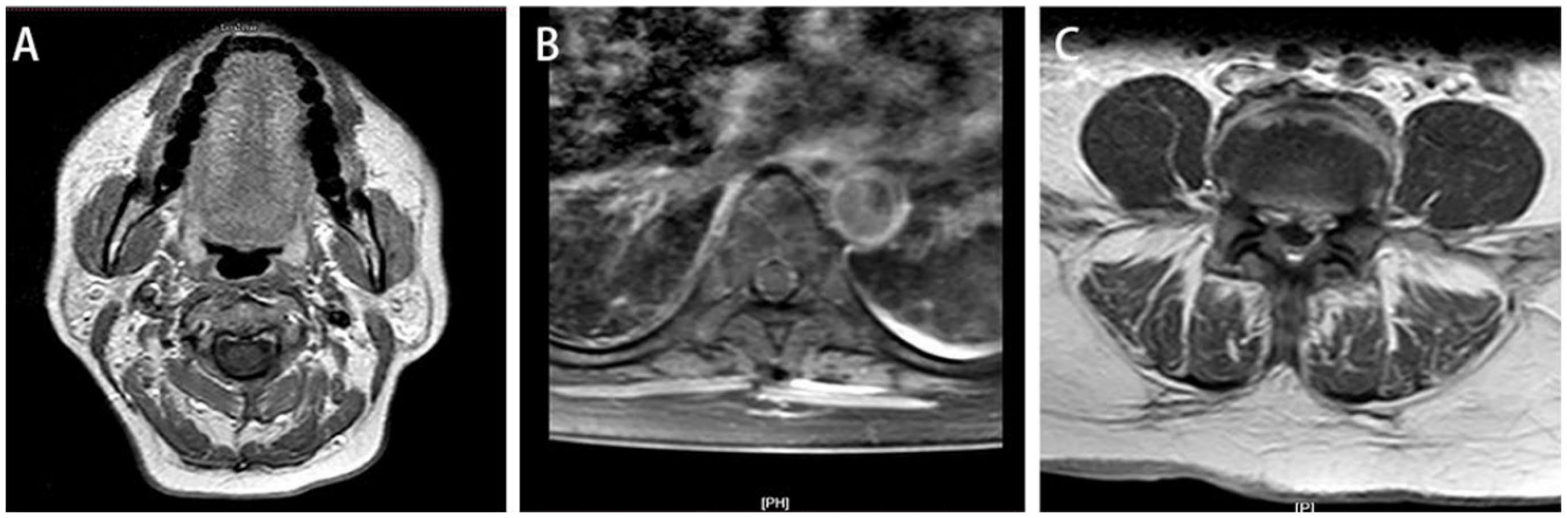
At 2-month follow-up, sagittal T1-weighted **(A)** and T2-weighted image **(B)** and showed the lumbar disc herniation was still present, and the epidural space in the posterior lumbar and anterior sacrococcygeal segments was wider than before, and the enhancement of the dural sac improved after enhancement compared with before. Axial T1-enhanced image **(C)** showed the previously large range of irregular non-enhanced areas has decreased compared to Figure 1F.

**Figure 3:**
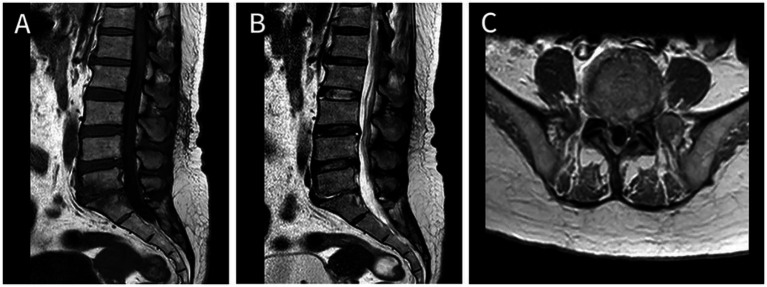
At 1.5-year follow-up, Axial T1-enhanced image of the cervical **(A)**, thoracic **(B)**, and lumbar **(C)** segments demonstrated that the epidural abscess was almost completely absorbed, and the epidural space now shows normal signal intensity, with a largely improved corresponding spinal cord compression.

A 52-year-old Chinese female patient was admitted to the emergency department due to 2 years of low back pain and 3 days of decreased muscle strength in the extremities. 2 years ago, she began experiencing lumbar back pain with mild lower limb radiating pain and numbness. After she underwent home physical therapy 1 week ago, the aforesaid symptoms intensified, and she went to the local hospital for an MRI of the lumbar spine, which revealed lumbar disc herniation. Three days ago, she started experiencing significant back pain and dysuria, followed by abnormal muscular strength and hypoesthesia in both lower limbs the next day. A day after that, the symptoms spread to both upper limbs. The patient had a history of Total Knee Arthroplasty (TKA) 6 years prior, which was performed under combined spinal-epidural anesthesia, with no reported history of diabetes or other diseases. Upon physical examination, the patient exhibited hypoesthesia below the inguinal plane, as well as biceps, triceps, finger flexors, and finger extensors muscle strength of grade 3 on both sides, muscle strength of grade 1 in both lower limbs, no fecal incontinence, and intact rectal tone. Upon admission, the patient presented with shortness of breath and confusion. A large amount of light red, thick sputum was aspirated by sputum suction. After face mask oxygenation, oxygen saturation was around 80%. Therefore, the patient was transferred to the intensive care unit (ICU) due to type II respiratory failure and a maximum temperature of 40.8°C. Blood tests showed elevated infection markers, including a total white blood cell (WBC) count of 26.6 × 10^9^/L (normal range: 3.5–9.5 × 10^9^/L), 85% neutrophils (normal range: 40–75%), increased C-reactive protein (CRP) levels of 25.03 mg/dL (normal range: <0.6 mg/d L), and increased procalcitonin (PCT) levels of 1.96 ng/mL (normal range: <0.05 ng/dL). Although a fluoroscopic-guided lumbar puncture was unsuccessful, an intravenous gadolinium-enhanced magnetic resonance imaging (MRI) revealed an abnormal signal shadow ventral to the spinal canal in the cervicothoracic segment and dorsal to T12-S1 level (Figure 1). Blood culture results confirmed the presence of *S. intermedius*, which was sensitive to vancomycin. Unfortunately, we did not rule out the possibility of false-positive blood cultures by means of genetic tests such as 16S rRNA or mNGS. Empirical IV antibiotic treatment with vancomycin (50u ivd q8h) was initiated, followed by the addition of meropenem (1 g ivd q8h) and moxifloxacin (0.4 g ivd qd) after receiving the blood culture results. Vancomycin dosing was adjusted dynamically based on blood concentration. The patient’s clinical presentation of paraplegia and decreased muscle strength did not improve during the first 2 days of initial antibiotic treatment. The spine surgeons, in conjunction with the ICU physicians, discussed the following results: The patient’s rapidly progressing and worsening neurologic symptoms within 72 h require urgent decompression surgery. However, the cervicothoracic segment abscess located ventrally may not drain adequately. Abscesses of the lumbar segment located dorsally have relatively poor outcomes after decompression surgery, and there is no additional benefit to early surgical treatment. Patients may not tolerate the shock of prolonged decompressive surgery of multiple spinal segments. Therefore, the patient was informed that progressive neurological deterioration may not be completely resolved even after delayed surgical treatment. After obtaining informed consent, we decided to continue treatment with antibiotics for at least 6 weeks. After 5 days of IV antibiotic treatment, blood culture results were negative. Following 1 month of antibiotic treatment, the patient’s maximum temperature decreased to below 38.0°C, and her WBC count was 10 × 10^9^/L with a CRP level of 6.19 mg/dL. After 43 days of IV antibiotic treatment, the patient exhibited grade 4 muscle strength bilaterally in the biceps, triceps, finger flexors, and finger extensors. At this time, a repeat MRI showed a significant reduction in the holospinal abscess, particularly in the upper lumbar segment (Figure 2). After 45 days of IV antibiotic treatment, the patient was transferred back to the general ward from the ICU. Laboratory examinations after 50 days of IV antibiotic treatment demonstrated normal findings in the patient’s WBC count, neutrophil count, CRP level, and PCT level. However, despite 2 months of hospital treatment, there was no significant improvement in weakness observed in both lower extremities. At the 1.5-year follow-up, a repeat MRI revealed complete resolution of the epidural abscess (Figure 3), and the patient’s muscle strength returned to grade 5 in the extremities. Nevertheless, numbness in both lower extremities persisted.

## Discussion

### Physiopathology

The anterior epidural space is mostly occupied by the dura, posterior longitudinal ligament, and periosteum of the vertebral body, which are closely adherent. Hence, SEA occurs in the posterior epidural space (4). The etiopathogenic mechanism of spinal involvement in our patient remains to be determined. However, our case is unique as the cervicothoracic abscess occurred in the anterior epidural space, while the lumbosacral abscess occurred in the posterior epidural space, suggesting a possible route of infection. Tetsuka et al. (5) believed that SEAs secondary to septic spondylitis or intervertebral discitis tend to be located anterior to the dural tube, while those due to hematogenous infections tend to be located posterior to the dural tube. The vertical sheath of the epidural space enables the spread of abscesses from the level of origin to multiple levels longitudinally. Infections can enter the epidural space through various mechanisms, including hematogenous spread (50%), direct extension from adjacent infection (33%), inoculation from spinal procedures (15%), and unknown mechanisms (6, 7). The incidence of infection after combined spinal-epidural anesthesia is approximately 1/2000 (5, 6). Other potential risk factors include diabetes, human immunodeficiency virus (HIV) infection, trauma, tattooing, acupuncture, and infection of the adjacent bone or soft tissue (8, 9). Additionally, degenerative disk disease, large osteophytes, and chronically hypertrophied facet joints may be targets of hematogenous spread (10). Hematogenous spread is commonly caused by methicillin-resistant *Staphylococcus aureus* (MRSA), and the exotoxins, extracellular enzymes, and cell surface substances of MRSA can cause ischemic changes and tissue destruction in the spinal cord through various mechanisms, ultimately leading to spinal cord injury (11). Other bacteria causing hematogenous spread include negative cultures (13.9%), Gram-negative bacteria (8.1%), coagulase-negative *staphylococci* (7.5%), and *Streptococcus anginosus* group (SAG, 6.8%) (12).

Blood culture following an admission of our patient indicated *S. intermedius*. *S. intermedius* is one of the three species found in the SAG, also known as the *Streptococcus milleri* group (SMG) (2). SAG is a commensal in the oral and gastrointestinal tracts. But SAG is notorious for causing invasive infections, including head and neck abscesses, bacteremia with endocarditis, liver abscess, thoracic empyema, brain abscess, and spinal epidural abscess (13, 14). Although rare, patients with underlying diseases such as cirrhosis, diabetes, and malignancy are susceptible to SAG infection (15). We reported a case of an extensive SEA treated conservatively, along with a review of relevant literature and discussions on SEA caused by *S. intermedius*. We conducted a PubMed search using the keywords “*Streptococcus anginosus* group abscess” and “*Streptococcus intermedius* epidural abscess,” to identify reports and studies on spinal abscess caused by *S. intermedius*. Only 6 cases were found, including the present case, with an average patient age of 61 (standard deviation: 18.01) years, and four of the seven patients were women. In contrast to the case report mentioned in Table 1 (14, 16–20), this patient had no known risk factors such as dental disease, surgery, or trauma. After 2 months of IV antibiotic treatment, the patient showed partial resolution of the epidural abscess on MRI. Gangone et al. (21) reported a case of an immunocompetent 72-year-old patient with a complaint of simple low back pain, who was found to have co-infection with *S. intermedius* and *Streptococcus griseus*. The patient was treated with IV antibiotics and recovered completely. Although Gangone et al. (21). found no such dental intervention in their patient’s medical history, poor dental hygiene is a known predisposing factor for spinal abscesses and infective endocarditis. Similarly, our patient was immunocompetent, only had lumbar disc herniation and lumbar spondylolisthesis located at L5 level. Although we have not yet determined the exact cause of the patient’s extensive SEA, the combination of imaging and medical history suggests that multiple confounding factors may have contributed to the condition. We believe this case highlights the importance of considering *S. intermedius* as a potential causative agent of SEA, particularly in patients with underlying diseases.

**Table 1:** Review of spinal epidural abscess (SEA) cases with pathogenic reports of *Streptococcus intermedius* (*S. intermedius*) infection.

Authors/year of study (reference)	Age	Sex	Predisposing factors	Symptoms	Affected levels	Surgery	Outcome	Following-up duration (months)
Lee et al., 2022 (16)	68	M	Diabetes Chronic periodontitis	5-day fever and back pain urine retention paraplegia	T2 to T4 L1 to S3	Laminectomy Discectomy	Return to daily activities	17
Ramhmdani and Bydon, 2017 (17)	74	M	root canal procedures	Urine retention Paraplegia	L1 to L5 T10 to T11	Laminectomy Surgical drainage	Resolution of abscess on MRI	3
Heckmann and Pauli, 2015 (18)	77	F	Single-tooth extraction	5-day neck stiffness	C1 to T7	Neurosurgical therapy	Recovery	NR
Lampen and Bearman, 2005 (14)	25	F	Pregnancy	2-week Shoulder pain 3-day Urine retention 3-h Paraplegia	T1 to T2	Laminectomy	Return to daily activities	10
Yang et al., 2018 (19)	67	F	percutaneous intradiscal injection	4-day Urine retention 4-day lower limbs numbness	C2 to T1	Surgical drainage	MRI abnormalities	12
Shiu et al., 2014 (20)	69	M	Diabetes	1-week fever Paraplegia	T4 to T8 L2 to L4	laminectomy vertebroplasty surgical drainage	Failed to recovery	2

M, male; F, female; NR, Not Referring.

### Clinical presentation

SEA is typically characterized by the triad of fever, back pain, and neurological dysfunction. However, only 0.8% of patients exhibit this classic presentation on admission, making the diagnosis of SEA challenging (22). The prevalence of back pain and the rarity of SEA create a diagnostic conundrum, resulting in delayed diagnosis in 75–89% of cases (23). In this case, the patient presented with typical symptoms, progressing through four stages of back pain, nerve root symptoms, muscle weakness, and paresthesia, ultimately leading to complete paralysis within 1 day of entering the third stage. This progression is consistent with Heusner’s description of the four progressive stages of clinical presentation of SEA, which are variable in duration and may result in unpredictable neurological deterioration, underscoring the importance of prompt diagnosis and treatment (24). Although abnormal laboratory values such as leukocytosis or elevated inflammatory markers and isolation of pathogenic pathogens from blood cultures may be predictive of disease severity in established diseases, they are not specific for diagnosis (25). Furthermore, in this case, the patient’s MRI performed before admission did not reveal SEA, further highlighting the diagnostic challenge associated with this condition.

### Imaging diagnosis

In suspected cases of SEA, imaging should be promptly performed. While lumbar puncture results showing perimembranous inflammation can aid in the diagnosis, this method is invasive and carries the risk of spreading the infection to the subarachnoid space or causing meningitis, as is the case with CT myelography (26). Therefore, after two lumbar punctures were performed and failed, we did not attempt a third. Puncture failure may be caused by the patient’s obesity (27, 28). MRI is the most accurate diagnostic tool for SEA, revealing not only the presence and extent of the abscess but also the degree of spinal cord compression. However, MRI findings in the early phase of the clinical course can be insignificant or subtle. Typical features of spinal infections include the contiguous involvement of two vertebrae and inflammatory changes within the intervertebral disc, but these are relatively chronic changes that may take weeks or months to manifest (29). A diagnostic delay of 4 months is not uncommon, given the time lag between initial symptoms and MRI (30). Intravenous gadolinium-enhanced MRI has high sensitivity and specificity, allowing for the diagnosis of SEA as epidural masses with surrounding septic or necrotic material exhibiting linear enhancement on T1-weighted contrast-enhanced MRI or hyperintensity on T2-weighted MRI (31). In cases where the diagnosis is still unclear after enhanced CT and plain MRI, a gadolinium-enhanced MRI should be performed, as recommended by Dunbar et al. (30) He reported a case of an immunocompetent patient who underwent two enhanced CT and one plain MRI with no results until undergoing enhanced MRI, which provided a clear diagnosis of SEA due to *Pasteurella multocida* (30). In our case, The patient presented with neurological root symptoms but no fever before admission. Initial MRI and plain radiography diagnosed the patient with simple lumbar disc herniation, which may have been either underdiagnosed or caused by a rapidly developing abscess in the week, leading to subsequent neurological impairment. Therefore, repeat examinations or gadolinium-enhanced MRI should be considered when initial MRI findings are not diagnostic of SEA.

### Management

For SEA confirmed by imaging, the standard treatment has traditionally been urgent surgical decompression followed by 6 weeks of IV antibiotic therapy. The literature suggests that surgical intervention is necessary in cases of acute or progressive neurological deficits, spinal instability, progressive deformities, or disease progression despite antibiotic therapy (7). Kim et al. (32) identified four independent predictors of nonoperative management failure in a cohort of 142 medically managed patients: age > 65 years, diabetes, neurologic impairment, and MRSA. If all four risk factors are present, they report a 99% failure rate. In a similar study, Patel et al. (33)reported three additional independent predictors of failure: leukocytosis >12.5, positive blood cultures, and CRP >115. If all three risk factors are present, they report a 77% failure rate (33). However, urgent surgical decompression is not always beneficial, as it may result in significant surgical trauma, impair spinal stability, and require spinal fusion surgery, which can reduce the patient’s range of motion in the lower back (34). The timing of surgical intervention for SEA is also controversial (35). In this case, we considered the patient’s poor condition and concluded that open surgery involving multiple segments may not be well-tolerated, despite the presence of independent predictors of non-surgical treatment failure and a high rate of non-surgical treatment failure as reported by Kim and Patel. The epidural compression was primarily caused by granulation tissue, and the size of the epidural abscess was relatively small. Therefore, we determined that excising only a small portion of the granulation tissue and performing decompression would not be particularly beneficial (36). Additionally, the origin of the abscess, whether it is ventral or dorsal, affects spinal stability after decompression surgery. Karikari et al.’s (37) regression model showed that ventral abscesses can be approached ventrally, preserving the posterior longitudinal ligament and reducing postoperative morbidity. We believed that this patient’s lumbar abscess originated from the dorsal side and had a relatively poor prognosis compared to the ventral side.

MRI is a crucial diagnostic tool for SEA patients as it provides accurate and objective imaging, enabling conservative treatment options consisting of systemic antibiotics and CT-guided percutaneous needle aspiration. Adogwa et al. (38) examined 82 cases of SEA in patients over the age of 50 with multiple comorbid conditions and found that early surgical decompression combined with IV antibiotics was not superior to IV antibiotics alone in this small group of patients. Arko et al. (12) came to similar conclusions. He concluded that patients can usually be treated with intravenous antibiotics and do not always require surgery, even though the patient may deteriorate clinically at any time (12). Antimicrobial agents are best chosen for the causative organism identified in blood cultures. Without knowing the causative bacteria, treatment should be initiated empirically. However, it is likely that different diseases, such as spinal abscess, liver abscess, and cystic fibrosis, will display significant differences in antimicrobial susceptibilities relative to published reports, given the frequent exposure of patients with underlying diseases to chronic macrolide suppressive therapy, inhaled aminoglycosides, and frequent use of fluoroquinolones (39). The previous standard antibiotic regimen for the treatment of abscesses associated with *S. intermedius* was penicillin plus chloramphenicol, but increasing resistance of *S. intermedius* to some antibiotics and the possibility of antagonism between penicillin and chloramphenicol have been documented (2). The most potent empiric therapies for *S. intermedius* include vancomycin, teicoplanin, and imipenem (40, 41). The British Society of Antimicrobial Chemotherapy (BSAC) advises that abscesses may be treated with a combination of Cefotaxime (a beta-lactam antibiotic) and metronidazole parenterally for 3 to 4 weeks or between 4 and 6 weeks when they are aspirated (42). Our case involved a patient with extensive SEA caused by *S. intermedius* infection, who presented with type II respiratory failure upon admission and was immediately transferred to the ICU for tracheal intubation. She had no sinusitis, heart disease, dental procedures, or other known risk factors for abscesses caused by *S. intermedius*. Intravenous vancomycin was initiated before the results of the blood culture were available, and after the culture results became available, meropenem and moxifloxacin were added. Fortunately, the patient’s drug sensitivity test results suggested sensitivity to these antibiotics. The duration of antibiotic administration is supported by the literature. After 5 days of antibiotic therapy, the blood culture became negative, and a repeat MRI performed 6 weeks later revealed a decrease in abscess size compared to the previous scan. After 2 months of antibiotic treatment, the patient’s muscle strength was restored. These outcomes suggest that our conservative treatment approach was successful in managing the patient’s condition.

## Conclusion

SAG is a small contributor to spinal infections. However, neurological symptoms associated with SAG can deteriorate rapidly, with nerve root symptoms progressing to extremity paralysis in as little as 1 day. The classic triad of fever, back pain, and neurological dysfunction should prompt immediate suspicion of SEA, and a prompt repeat general MRI or intravenous gadolinium-enhanced MRI should be performed, even if the initial MRI was negative. Empirical therapies that have shown efficacy against *S. intermedius* include vancomycin, tacrolimus, and imipenem. Given our patient’s circumstances involving multiple segmental spinal abscesses and respiratory failure, the timely and appropriate administration of sensitive IV antibiotic therapy may also be an effective approach in treating extensive SEA.

## Data availability statement

The original contributions presented in the study are included in the article/supplementary material, further inquiries can be directed to the corresponding author.

## Ethics statement

Ethical review and approval was not required for the study on human participants in accordance with the local legislation and institutional requirements. The patients/participants provided their written informed consent to participate in this study. Written informed consent was obtained from the individual(s) for the publication of any potentially identifiable images or data included in this article.

## Author contributions

DL conducted the data analysis and wrote the first draft. WL critically revised the article. WH and WZ rearranged the figures and enriched the interpretation of the figures. QL was directly responsible for the manuscript. All authors made significant contributions to the article’s conception and design.

## Conflict of interest

The authors declare that the research was conducted in the absence of any commercial or financial relationships that could be construed as a potential conflict of interest.

## Publisher’s note

All claims expressed in this article are solely those of the authors and do not necessarily represent those of their affiliated organizations, or those of the publisher, the editors and the reviewers. Any product that may be evaluated in this article, or claim that may be made by its manufacturer, is not guaranteed or endorsed by the publisher.
